# Boosting the Therapeutic Potential of Extracellular Vesicles Derived From Mesenchymal Stem Cells via Advanced Preconditioning for Neurodegenerative Disorders

**DOI:** 10.1155/sci/2616653

**Published:** 2025-08-21

**Authors:** Cristina D'Arrigo, Sara Labbate, Denise Galante

**Affiliations:** Institute of Chemical Sciences and Technologies “Giulio Natta”, National Research Council of Italy, Genoa, Italy

**Keywords:** extracellular vesicles, mesenchymal stem cells, neurodegenerative disorders, preconditioning methods

## Abstract

Acute and chronic neurodegenerative conditions (NDs) are major causes of disability and mortality worldwide. Acute NDs encompass conditions such as stroke, traumatic brain injury (TBI), and spinal cord injury (SCI). On the other hand, chronic NDs include Alzheimer's disease (AD), Parkinson's disease (PD), Huntington's disease (HD), multiple sclerosis (MS), and amyotrophic lateral sclerosis (ALS). Currently, no definitive cure exists for these diseases, and available therapies focus primarily on slowing the progression of symptoms. Mesenchymal stem cells (MSCs), due to their multilineage differentiation capacity, immunomodulatory abilities, and regenerative properties, have gained attention in regenerative medicine. In recent years, extracellular vesicles (EVs) derived from MSCs have shown great promise as a cell-free therapeutic approach, eliminating the risks associated with direct MSCs use, such as tumorigenicity and poor cell survival after transplantation. EVs have emerged as powerful mediators of intercellular communication and tissue repair, exhibiting immunomodulatory, anti-inflammatory, and proregenerative properties. However, limitations such as low EVs yield and reduced efficacy due to MSCs replicative senescence restrict their therapeutic potential. Preconditioning strategies, including hypoxia, 3D cultures, and biochemical priming, have been explored in other fields to enhance EVs properties, yet their specific application to NDs remains under-reported. This review aims to address this gap by analyzing the preconditioning methods used to boost the therapeutic potential of MSCs-derived EVs for neurodegenerative diseases. These preconditioning strategies may enhance EVs yield, functional cargo, and targeted therapeutic efficacy for treating acute and chronic NDs.

**Summary**

• MSCs-derived EVs modulate inflammation and promote neuro-regeneration.
• Challenges: Low yield of EVs and reduced efficacy due to MSCs replicative senescence.
• Preconditioning boosts the therapeutic potential of MSCs-EVs in neurodegenerative diseases.
• Hypoxia, cytokine, animal brain extracts, and 3D culture enhance EVs yield and potency.
• Preconditioning methods optimize EVs properties for enhanced neuroprotection.

## 1. Introduction

Neurons are fundamental to brain function, facilitating communication throughout the body. Neurodegeneration, the progressive loss of neurons and glial cells in the brain and/or spinal cord underlies various brain disorders collectively termed chronic neurodegenerative conditions (NDs). NDs, including Alzheimer's disease (AD), Parkinson's disease (PD), Huntington's disease (HD), multiple sclerosis (MS), and amyotrophic lateral sclerosis (ALS), affects millions worldwide, with age, genetic, and environmental risk factors.

In AD, the accumulation of amyloid beta (Aβ) and tau proteins leads to plaque formation, disrupting brain circuitry, and causing memory impairment and other cognitive deficits [1, 2]. PD is characterized by the loss of dopamine-producing neurons, resulting in motor symptoms and various nonmotor manifestations. Moreover, α-synuclein misfolding and aggregation, mitochondrial dysfunction, and neuroinflammation contribute to PD pathophysiology [3, 4]. HD is a hereditary NDs that affects muscle coordination and leads to cognitive decline and psychiatric problems. It is caused by a mutation in the HTT gene, which produces an abnormal form of the protein huntingtin (Htt)[5, 6]. ALS, or Lou Gehrig's disease, involves progressive motor neuron deterioration, leading to muscle weakness and paralysis. Genetic mutations and intraneuronal protein aggregates, including TAR-DNA binding protein, are implicated in ALS pathogenesis [7]. MS is an autoimmune NDs and is characterized by inflammation with demyelination, astroglial proliferation (gliosis), and neurodegeneration [8].

Moreover, acute neuronal injuries such as stroke, traumatic brain injury (TBI), and spinal cord injury (SCI) induce secondary neurodegeneration [9–12]. Stroke causes progressive loss of tissue in the area damaged by infarction, after SCI, the death of neurons and the breakage of neuronal axons cause dysfunction of neural circuits, and finally TBI is a risk factor for AD, PD, and ALS, because the traumatized tissue may induce chronic inflammation that can promote neurological impairment and neurodegeneration [13, 14].

While there is currently no cure for most NDs, several therapies aim to alleviate symptoms, slow disease progression, and improve quality of life. Over the past few decades, mesenchymal stem cells (MSCs) have garnered significant attention in the field of regenerative medicine, including their potential therapeutic applications for NDs. MSCs are versatile adult stem cells capable of differentiating into various mesoderm-derived cell types. Derived from either embryonic or adult sources, such as bone marrow (BM), amniotic fluid (AM), adipose tissue (AT), dental pulp (DP), and umbilical cord (UC), MSCs possess two critical characteristics: [15] multilineage differentiation potential and self-renewal capacity. With their inherent trophic, immunomodulatory, proregenerative, anti-inflammatory, proangiogenic, and antiapoptotic properties, MSCs offer immense potential in preclinical investigations for treating diverse medical conditions. Their ability to cross the blood-brain barrier (BBB) facilitates delivery to the central nervous system. Among the diverse repertoire of paracrine factors secreted by MSCs, extracellular vesicles (EVs) have emerged as key mediators of intercellular communication, tissue repair, and immunomodulation [16]. EVs are nanoparticles encapsulated by phospholipid bilayers, with a size range of 30–5000 nm, belonging to three categories: apoptotic bodies (ranging from 500 to 5000 nm), microvesicles (ranging from 100 to 1000 nm), and exosomes (ranging from 30 to 150 nm). In particular, exosomes contain a variety of molecules, including proteins, lipids, nucleic acids (such as miRNAs, mRNAs, and DNA), and metabolites. The cargo composition of exosomes can vary depending on the cell type and physiological state [17].

As many studies reported, MSCs-derived EVs have become a ground-breaking, cell-free approach that bypasses difficulties related to the direct use of MSCs, such as aging, possible tumor formation, low engraftment, and low quantities to target tissue due to the BBB passage [18–20]. At the same time, EVs retain some characteristics of their parent MSCs [21], such as immune system modulation, regulation of neurite outgrowth, promotion of angiogenesis, and the ability to repair damaged tissues [22].

Additionally, EVs can be used as drug carriers, exploiting their biocompatibility, small size, and ability to transport various molecules. Despite the vast potential of EVs, there are still many limitations to their use. These include: (1) the low yield of EVs obtained from MSCs; (2) the replicative senescence of MSCs after multiple in vitro passages which reduces the therapeutic efficacy of their secreted EVs in vivo; (3) difficulties in determining an optimal therapeutic treatment regimen; (4) the rapid clearance of MSC-derived EVs by the reticuloendothelial system, including the liver, spleen, and other immune organs, leading to poor targeting to injury sites in vivo [23]. The EVs composition, therapeutic efficacy, and functional properties can be influenced by the microenvironment and physiological conditions during their biogenesis and release [24]. Preconditioning strategies aimed at modulating the cellular milieu and priming MSCs before EVs isolation have emerged as a promising approach to enhance the yield, potency, and therapeutic cargo of EVs [25].

These strategies include a diverse array of modalities, including biochemical, physical, and pharmacological interventions such as three-dimensional cultures, exposure to hypoxia, and the utilization of various biochemical signals. They can upregulate key regenerative and immunomodulatory factors in MSCs, which are subsequently enriched in the EVs cargo, including growth factors, microRNAs, and cytokines, thereby, improving their therapeutic efficacy. Furthermore, preconditioning enables the tailoring of EVs content to better mimic physiological or pathological conditions, enhancing their clinical relevance. In addition to improving yield and therapeutic efficacy, preconditioning strategies can also enhance the stability, targeting ability, and in vivo persistence of EVs. For instance, hypoxic preconditioning has been shown not only to increase the secretion of EVs but also to enrich them with specific miRNAs and proteins that confer enhanced neuroprotective, antiapoptotic, and anti-inflammatory effects [26]. Similarly, 3D cultures and biomimetic scaffolds more closely replicate the physiological environment of MSCs, promoting the production of EVs with properties better suited to the treatment of complex tissues like the brain [27]. Moreover, pharmacological preconditioning with small molecules or cytokines can selectively modulate EV cargo composition to address specific pathological features of neurodegenerative diseases, such as oxidative stress or mitochondrial dysfunction. Emerging approaches also explore the combination of multiple stimuli (e.g., hypoxia with inflammatory cytokines) to synergistically boost the therapeutic payload of EVs [28, 29]. Importantly, these strategies may also influence the surface molecules of EVs, enhancing their capacity to evade immune clearance and improving their biodistribution and homing abilities [30]. Thus, preconditioning not only represents a tool to potentiate EV-based therapies, but also a step toward greater customization and precision in regenerative medicine applications. Unlike nonpreconditioned MSC-EVs, which can vary due to donor differences and culture conditions, EVs derived from preconditioned MSCs provide a more standardized and reproducible therapeutic product, enhancing their reliability for clinical applications [23].

**Figure 1:**
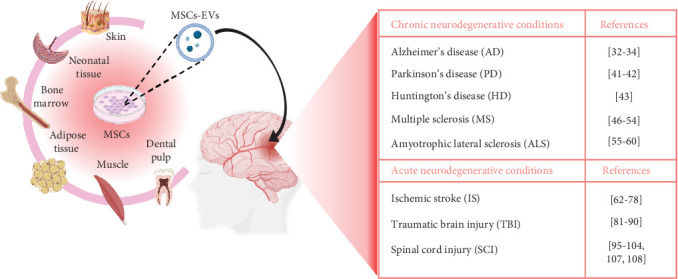
Representation of sources of MSCs-EVs and summary of studies on their role in NDs.

So far, in the literature, there are few protocols for preconditioning MSCs applied to NDs with descriptions of the effects on derived vesicles on in vitro and in vivo models. This paper provides a comprehensive review of preconditioning strategies employed to improve the properties and functionality of EVs derived from MSCs in acute and chronic neurodegenerative diseases. We first summarized the application of MSCs-derived EVs in different NDs (Figure 1). Besides, we discussed different types of preconditioning and the resulting impacts on the therapeutic use of preconditioned MSCs-derived EVs in cellular and animal models.

## 2. MSC-Derived EVs Applications in Chronic and Acute NDc

### 2.1. AD, PD, and HD

EVs derived from MSCs contain bioactive molecules, including growth factors, cytokines, microRNAs, and other regulatory RNAs, which can modulate neuronal function, reduce inflammation, and promote neuroprotection and synaptic plasticity, holding promise as a potential treatment strategy for NDs such as AD, PD, and HD (Table 1).

#### 2.1.1. AD

The application of EVs derived from various sources, particularly human umbilical cord mesenchymal stem cells (hUC-MSCs-exosomes) and human bone marrow mesenchymal stem cells (hBM-MSCs-EVs), had a significant impact on AD therapy [31].

In a recent study, hUC-MSCs-exosomes injected into the tail veins of APP/PS1dE9 mice, increased anti-inflammatory cytokines, and decreased proinflammatory cytokines in both peripheral blood and brains [31]. Furthermore, they reduced Aβ levels upregulating the expression of insulin-degrading enzyme (IDE) and neprilysin (NEP), and improved spatial learning and memory decline in AD mice.

Additional evidence showed that hUC-MSCs-EVs support the therapeutic efficacy on the neuronal deficits in Aβ-stimulated hippocampal neurons in both cells and animal models [32]. Behavioral assessments revealed that hUC-MSCs-EVs treatment improved learning, memory, and recognition in APP/PS1 mice, while reducing Aβ-plaques, hippocampal neuronal loss and their structural impairments. Investigating the early stage of the disease, hBM-MSCs-EVs promoted the Aβ plaque disaggregation in 5-month-old APP/PS1dE9 AD mice and prevented new plaque formation in 3-month-old mice, likely via direct interaction with Aβ plaques and delivery of Aβ-degrading enzymes [33].

ASC-derived EVs, enriched with neuroprotective proteins like Filamin-A and RhoGDI1, promoted neurite growth and neurogenesis in APP/PS1 mice, after IN administration [34]. In addition, MSCs-EVs also appear to positively influence the interaction between neurons and microglia, reducing microglial activation in AD mouse models.

Although the mechanism is not yet fully understood, hBM-MSCs-EVs improve AD symptoms in AD rat model by delivering miR-29c-3p to neurons, targeting BACE1 gene, and activating the Wnt/β-catenin pathway, with anti-inflammatory and neuroprotective properties [35, 36]. Moreover, as hUC-MSCs-EVs, the treatment significantly improved AD rat behaviors, reduced Aβ deposition and Aβ1-42 levels, increasing NEP and IDE expressions. hBM-MSCs-EVs also enhanced neuron viability, reduced apoptosis, and inhibited Aβ plaque, while exosomes isolated from human Wharton's jelly MSCs (hWJ-MSCs-exosomes), promoted Aβ degradation, improved brain glucose metabolism/cognitive function, and modulated the epigenetics and genetic expression in AD models [37]. Intracisternal administration of iPSC-MSCs-small EVs (sEVs) in a sporadic mouse model of AD [38], reduced NLRP3/GSDMD-mediated neuroinflammation, decreased amyloid deposition and neuronal apoptosis, and alleviated cognitive dysfunction. It was found that miR-223-3p directly targeted NLRP3, and that inhibiting miR-223-3p almost completely reversed the suppression of NLRP3 by MSCs-sEVs, suggesting that miR-223-3p may play a significant role in MSCs-sEVs-mediated anti-inflammatory effects [39].

#### 2.1.2. PD and HD

Exosomes derived from hUC-MSCs protected dopaminergic neurons in PD [40]. These exosomes exhibited the ability to enhance cell viability, reduce levels of apoptosis-related proteins, and decrease apoptotic rates in 6-hydroxydopamine (6-OHDA)-stimulated SH-SY5Y cell proliferation by inducing autophagy. Given the dopaminergic neuron loss in the substantia nigra in PD, the ability of exosomes to cross the BBB and protect neurons in the substantia nigra is therapeutically promising. In PD rats, exosome treatment reduced motor asymmetry, likely via transfer of proteins, mRNAs, and miRNAs (e.g., miR-133b) to target cells [40].

Bai et al. [41] studied EVs from hUC-MSCs containing miR-106b in a PD animal model induced by neurotoxin 1-methyl-4-phenyl-1,2,3,6-tetrahydropyridin (MPTP). The MSCs-EVs-miR-106b promoted autophagy and mitigated apoptosis in neurons affected by PD. The neuroprotective effect was demonstrated both in vitro and in vivo by downregulating CDKN2B. EVs containing miR-106b help rescue neurons, enhance autophagy, and inhibit apoptosis.

The neuroprotective potential of hASC-derived EVs, explored in HD in vitro models [42], demonstrated beneficial effects in slowing HD progression, improved mitochondrial function, modulated apoptotic proteins, and enhanced cell survival. Effects included activation of the p-CREB-PGC1 pathway, and reduction of mutant huntingtin (mHtt) aggregates.

### 2.2. MS and ALS

MSCs and their secreted EVs have emerged as potential therapeutic agents also in NDs characterized by demyelination and neuroinflammation. This section highlights several studies showcasing the promising therapeutic effects of MSCs-EVs in conditions like MS [43] and ALS (Table 2) [59].

#### 2.2.1. MS

Treatment with hBM-MSCs-EVs of two mouse models of demyelination (experimental autoimmune encephalomyelitis-EAE and cuprizone-induced demyelination) showed positive outcomes by enhancing oligodendrocyte precursor cell differentiation, promoting remyelination, modulating microglial polarization, and suppressing pro-inflammatory responses in the CNS [44]. These effects were mediated by modulating immune cell activity, especially T-cell proliferation, and shifting microglial polarization toward the anti-inflammatory M2 phenotype via IL-10 and TGF-β induction [45]. EVs from human periodontal ligament MSCs exhibited anti-inflammatory effects by suppressing leukocyte infiltration and reducing pro-inflammatory factors such as IL-17, IFN-γ, IL-1β, IL-6, and TNF-α [46]. Treatment of EAE mice with hUC-MSCs-EVs reduced pro-inflammatory cytokines (IFN-γ, TNF-α, and IL-17) and increased anti-inflammatory ones (IL-10 and IL-4) [47, 48]. Furthermore, murine ASC-EVs ameliorated EAE pathogenesis, by blocking activated T cell adhesion to inflamed CNS vessels, while human ASC-EV reduced T cells proliferation, mean clinical scores, leukocyte infiltration, and demyelination in chronic MS models [49, 50]. High-dose of EVs from early gestational chorionic villus-derived MSCs (PMSCs-EVs) treatment in EAE animals, reduced DNA damage in oligodendrocytes and preserved myelin in the spinal cord, indicating neuroprotective effects. In vitro experiments indicated that PMSCs-EVs promoted the oligodendrocyte maturation, showing both protective and regenerative effects [51].

Although the systemic injection of mouse ASC-EVs primarily accumulated in other organs, intranasal (IN) administration of EVs was more effective than MSCs in alleviating clinical symptoms in EAE mice [52].

#### 2.2.2. ALS

Exosomes from mouse ASC showed therapeutic potential in the ALS murine model SOD1 (G93A), which overexpresses the human mutant *SOD1* gene [53]. The repeated intravenous (IV) or IN administrations of mASC-exo improved motor function, protected lumbar motoneurons, and targeted particularly brainstem motor regions SOD1 (G93A) mice. In NSC-34(G93A) cells, mASC-exo offers neuroprotection, by modulating apoptotic, reducing proapoptotic, and increasing anti-apoptotic proteins [54]. Proteomic analysis reveals mASC-exo contains 189 proteins implicated in key protective pathways, including stress response and PI3K-Akt signaling. These support the idea that targeting apoptotic pathways may benefit ALS treatment.

Bonafede et al. [55] demonstrated that mASC-exo, administered after oxidative stress, rescued NSC-34 motoneuron-like cells from apoptosis. Their effects may be due to miRNAs that inhibit apoptosis, regulate cell cycle, and enhance proliferation.

hASC-exo treatment reduced SOD-1 aggregation in the G93A ALS in vitro model [56] and countered ROS-induced mitochondrial damage [57]. hASC-exo restored mitochondrial function proteins like p-CREB and PGC-1α, improving complex I's contribution to ETS (erythroblast transformation specific) and coupling efficiency, as well as restoring mitochondrial membrane potential [57]. These effects suggest hASC-exosomes support oxidative phosphorylation in NSC-34 SOD1 (G93A) cells.

EVs from various stem cell sources have been tested in ALS patients. Crose [58] reported significant improvement in an advanced ALS patient treated with hBM-MSCs-EVs, including reduced tremors and spasms. The patient also showed increased muscle function and strength in both upper and lower extremities.

### 2.3. Stroke

A stroke occurs when the blood supply to a part of the brain is disrupted, causing damage [60]. The two main types of strokes are ischemic (from artery blockage) and hemorrhagic (from vessel rupture). Ischemic stroke (IS) represents ~ 85% of cases [61], so our analysis is on this variation.

The most utilized animal model is C57BL6 mice or adult Wistar/Sprague–Dawley rats with induced middle cerebral artery occlusion (MCAO). Additionally, fetal sheep (with UC occlusion) and rhesus monkeys have been employed (Table 3) [62–64].

A single IN administration of hASC-EVs restored vascularization in the peri-infarct zone and reduced infarct volume. It also improved long-term motor and behavioral outcomes after permanent IS in rats [61].

Doeppner et al. [65] reported that IV injection of hBM-MSCs or their EVs improved neurological recovery after focal cerebral ischemia in mice. Both MSCs and MSCs-EVs induce long-term neuroprotection and angiogenesis, but only MSCs-EVs modulate the peripheral immune response, preventing postischemic immunosuppression.

In a transient MCAO mouse model, hBM-MSCs-EVs downregulated levels of proinflammatory mediators, inhibited astrocytes and microglia activation, and maintained BBB integrity by delivering the noncoding RNA, miR-124, possibly acting via the peroxiredoxin 1 (PRX1) [66]. hBM-MSCs-EVs promote neurite remodeling, neurogenesis, and angiogenesis, as well as MSCs, but without the risks of emboli or uncontrolled cell replication linked to cell therapy [67]. Dumbrava et al. [68] demonstrated that repeated IV doses of hBM-MSCs-EVs promoted neurological recovery and brain remodeling in aged rats after MCAO, without affecting infarct volume. MSCs-EVs reduced macrophage infiltration in the periinfarct area helping restore motor-coordination.

Rat ASC-EVs reduced apoptosis and infarct volume in MCAO mice via EV-carried miR-22-3p, which inhibits KDM6B-mediated effects on the BMF/BMP2 axis [69]. Instead, rat BM-MSCs-EVs reduced neuronal apoptosis induced by cerebral ischemia-reperfusion injury through the phosphorylation of AMP-activated protein kinase (AMPK) and inhibition of JAK2/STAT3/NF-κB, key neuroprotective signaling pathways [70].

Caveolin-1 (Cav-1), a regulatory protein involved in several pathophysiological processes, including endocytosis, aggravates BBB permeability in acute IS. MSCs-EVs counteract Cav-1-mediated endocytosis of tight junction (TJ) proteins (ZO-1 and Claudin-5), reducing BBB leakage and infarction volume while enhancing neurological function [71].

The thrombolysis treatment of acute IS, using tissue plasminogen activator (tPA) can degrade the BBB, inducing hemorrhagic transformation, which counteracts thrombolytic efficacy and is associated with a poor prognosis and high mortality rate. MSCs-EVs alleviated hemorrhage by inhibiting astrocyte activation and mitigating the tPA-induced disruption of the BBB. Specifically, MiR-125b-5p delivered by BM-MSCs-EVs targeted Toll-like receptor 4 (TLR4) and inhibited nuclear transcription factor-kappaB (NF-κB) signaling in astrocyte, thereby, helping to preserve BBB integrity [72, 73].

Monkey BM-MSC-EVs have shown therapeutic potential in a nonhuman primate (NHP) model of cortical injury. In aged rhesus monkeys (*Macaca mulatta*), the administration of monkey BM-MSC-EVs improved fine hand motor function [62], reduced perilesional inflammatory microglia and myelin damage [74, 75], mitigated hyperexcitability, maintained excitation-inhibition balance in ventral premotor cortex neurons [63, 64], supported motor circuits reorganization, and promoted pyramidal neuron survival [76].

Preterm neonates are susceptible to perinatal hypoxic-ischemic (HI) brain injury. Investigation in an ovine fetal model have shown that in utero IV administration of hBM-MSCs-EVs partially protects against hypomyelination, but failed to prevent apoptosis and reduce cerebral inflammation [77]. This contrasts with previous results in which the neuroprotective effects of hBM-MSCs could be partially due to the anti-inflammatory capacities of MSCs [100]. Differences in immune-modulating properties among MSCs preparations and the unique ability of MSCs to respond to their environment, may explain why MSCs promote anti-inflammatory effects, unlike MSCs-EVs [77]. Despite this, EVs still show therapeutic potential, preserving baroreceptor reflex sensitivity and protecting against ischemia-induced white matter injury.

### 2.4. TBI and SCI

TBI and SCI represent two of the most acute devastating conditions affecting the CNS, with high morbidity and mortality. TBI, commonly caused by external trauma (e.g., accidents, falls, or sports-related injuries), leads to brain damage, varying neurological deficits from mild cognitive impairments to profound disabilities. On the other hand, SCI involved spinal cord trauma, causing partial or complete loss of motor, sensory, and autonomic function below the injury. Despite medical advances, individuals affected by TBI and SCI often face long-term disability and diminished quality of life.

#### 2.4.1. TBI

TBI poses a major challenge due to its lasting impact on brain function and the subsequent chronic neuroinflammation. One of the mechanisms by which activated microglia contribute to a chronic neuroinflammatory state of the brain is the activation of nucleotide-binding domain leucine-rich repeat and pyrin domain-containing receptor 3 (NLRP3) inflammasomes [101].

In acute TBI, IN administration of hBM-MSCs-EVs prevented the shift from acute to chronic neuroinflammation [78] by inhibiting NLRP3 inflammasome activation in microglia and reducing proinflammatory cytokine release. This treatment improved cognition and behavior, likely attributed to microRNAs carried by MSCs-EVs that transform proinflammatory microglia into noninflammatory phenotypes. Additionally, hASCs-exo enhanced sensorimotor functional recovery, reduced neuronal loss, and promoted neurogenesis in the hippocampus [79]. Zhang et al. [80] demonstrated that MSCs-EVs improved cognitive and sensorimotor functions without reducing cortical lesion volume. This treatment also promoted brain angiogenesis and neurogenesis while suppressing the activation of astrocytes and microglia/macrophages, indicating anti-inflammatory effects [81, 82]. Additionally, EVs derived from hWJ-MSCs reduced microglia-mediated neuroinflammation via TLR4 signaling in rats with perinatal brain injury [83]. Similarly, Amini et al. [84] emphasized the protective effects of clonal MSCs-EVs under nutrient deprivation stress post-TBI. These EVs showcased neuroprotective capabilities comparable to passive immunotherapy against cis p-tau, indicating potential benefits in reducing cystinosis and apoptosis.

Bambakidis et al. [85] underscored the influence of hBM-MSCs-EVs on the brain transcriptome, highlighting their impact on immune response, synaptic transmission, and neurodevelopmental genes. Upregulation of NEUROD6 and BDNF (Brain-derived neurotrophic factor) suggests potential for neurogenesis and neuronal survival. Moreover, these EVs suppressed neuroinflammation, reducing pro-inflammatory cytokines, and improving BBB integrity [85].

Exosome treatment has shown neuroprotective potential, reducing pro-inflammatory cytokines, inhibiting apoptotic markers, and increasing neurotrophic factors like BDNF, suggesting tissue preservation, and enhanced neural plasticity post-TBI [86]. Early EV administration attenuated neurologic injury and reduced brain lesion size [86, 87].

#### 2.4.2. SCI

SCI disrupts the connection between the rostral and caudal spinal cord segments, leading to life-threatening lesions and loss of motor, sensory, and autonomic functions [102]. The pathophysiology of SCI consists of two stages: primary and secondary injury [103]. Primary injury directly damages neurons and glial cells, which is an irreversible event [104]. Secondary injury follows, triggering extracellular matrix (ECM) impairment, inflammatory reaction, and oxidative stress [105]. Recent studies highlight the critical role of the NLRP3 inflammasome in the second injury phase of SCI [88].

Epidural fat-derived hMSCs-EVs (hEF-MSCs-EVs) modulated inflammatory responses, by downregulating NLRP1, and NLRP3 inflammasomes, showcasing neuroprotective effects, reduced tissue damage, and induced significant functional recovery in a rat SCI model [88]. hEF-MSCs-EVs treatment downregulated pro-inflammatory factors (IL-1β, IL-18, and TNF-α), known to exacerbate tissue damage and neuroinflammation post-SCI and reduced activated microglia expression. Similarly, BM-MSCs-Exos from mice reduced IL-1β, TNF-α, and IL-6 in a SCI mouse model, demonstrating their anti-inflammatory properties [89].

IV administration of hASCs-EVs improved locomotor function and tissue preservation post-SCI, benefiting the nervous system. However, dose-dependency differences suggest variability in the secretome profile of EVs [90].

IV injections of hUC-MSCs-EVs improved functional recovery [91]. Investigations into the mechanism revealed that hUC-MSCs-Exos shifted macrophages from a pro-inflammatory (M1) to an anti-inflammatory (M2) phenotype, thus, reducing inflammation, and activated the ERK1/2 pathway, promoting NSC proliferation and neural differentiation, but did not induce NSC migration to the injury site [91]. Additionally, hUC-MSCs-Exos alleviated inflammation and promoted SCI recovery by inhibiting the NF-κB/MAPK signaling pathway [92].

Direct injection of hUC-MSCs-EVs into the injury site of a rat SCI model demonstrated that EVs localized at the injury site for at least a week EVs, reducing spinal cord damage, inhibiting glial scarring and cavity formation, and improving motor function, aligning with previous studies [93].

Exosomes from human epidural AT-MSCs inhibited pro-inflammatory cytokine release, reduced apoptosis, and modulated the NLRP3 inflammasome, promoting neuroprotection and functional recovery after SCI. Biodistribution of exosomes reveals their accumulation in damaged spinal cord tissues, reducing inflammation and aiding tissue regeneration [94]. mRNA sequencing revealed significant gene changes related to the ECM, inflammation, and immune response following exosome treatment.

Intrathecal administration of hWJ-MSCs-EVs reduced inflammatory markers expression (caspase 1, IL-1β, IL-18, TNF-α) at both mRNA and protein levels in the injured spinal cord tissue [95]. EVs also inhibited glial scar formation by lowering GFAP expression, promoting tissue repair. Moreover, upregulation in Nestin, a marker for neuronal progenitor cells (NPCs), indicates EVs stimulate NPCs and potentially facilitate neural regeneration after SCI.

Repeated intrathecal administration of ASCs-EVs, in pigs-SCI model, improved long-term motor recovery, preserved neural tissue, reduced cavity formation, and promoted myelination and angiogenesis [96]. In a mouse model, hASCs-exo administered via the tail vein, mitigated neutrophil activation by inhibiting neutrophil extracellular traps (NETs) and reduced secondary inflammation after SCI. Restoration of motor function was linked to exosomal miR-125a-3p [97].

Ferroptosis, a form of programed cell death characterized by reactive oxygen species (ROS) and iron accumulation, differs from autophagy, pyroptosis, and apoptosis [106]. Increasing evidence links ferroptosis to SCI [107]. Recent studies show MSCs-Exos alleviate SCI by inhibiting ferroptosis in spinal neurons through the lncGm36569/miR-5627-5p/FSP1 pathway [98] and by inhibiting the IL-17 pathway [99].

## 3. Challenges in the Application of MSC-Derived EVs

The therapeutic application of MSC-EVs in NDs and beyond, remains constrained by several challenges that limit their large-scale clinical translation. One major limitation is the inherently low yield of MSC-EVs under conventional culture conditions [108]. Additionally, prolonged in vitro passaging induces MSC senescence, which significantly reduces the therapeutic efficacy of secreted EVs [109]. Also the limited targeting capability of systemically administered EVs, particularly after intravenous injection, is considered an intrinsic property of native or unmodified exosomes [110]. Moreover, EVs functional integrity may be compromised under pathological conditions, where increased oxidative stress accelerates EVs degradation and activates cellular autophagy, further reducing their therapeutic potential [111].

Another challenge in the application of MSC-EVs is determined by the selection of the therapeutic dosage and the choice of the MSCs source from which the EVs are derived.

### 3.1. EVs Low Yield

One of the most critical bottlenecks in the clinical translation of MSC-derived EVs is their inherently low yield under standard culture conditions. This limitation affects not only the scalability of EV-based therapies but also their reproducibility and cost-effectiveness. The small quantities of EVs produced per unit of culture medium constrain the possibility of generating therapeutically relevant doses, particularly for systemic administration where large amounts are required to reach and affect target tissues. Furthermore, batch-to-batch variability in EVs concentration and composition becomes more pronounced when yields are low, complicating standardization efforts, and regulatory approval processes. Inadequate EVs availability can also delay experimental timelines and reduce the statistical power of preclinical studies, which often require repeated administrations. As a result, the low yield of MSC-EVs poses a significant hurdle for both basic research and therapeutic development, potentially undermining the clinical feasibility of these promising biological agents [112].

### 3.2. MSC Replicative Senescence

Replicative senescence of MSCs represents another major challenge in the production of functional EVs. As MSCs are expanded in vitro to generate sufficient cell numbers, they progressively lose their proliferative capacity and undergo phenotypic and functional changes. Senescent MSCs exhibit a senescence-associated secretory phenotype (SASP), which includes the release of pro-inflammatory cytokines, chemokines, and altered EVs cargo. This change in the secretome composition can significantly impair the therapeutic quality of the EVs produced, leading to diminished immunomodulatory, antiapoptotic, and regenerative effects. Additionally, senescence affects the homogeneity and consistency of EVs preparations, increasing variability between batches and potentially introducing undesired biological effects. EVs from senescent MSCs may also carry markers of cellular stress or damage, raising safety concerns in clinical applications. Consequently, the onset of MSC senescence during prolonged culture not only reduces the overall EVs yield but also compromises the functional integrity and safety profile of the EVs intended for therapeutic use [113].

### 3.3. MSC-EVs Therapeutic Dosage

The dosage of EVs represents a critical variable that must be carefully considered. Differences in the effective EVs dose may arise due to variations in isolation techniques, which influence EVs purity. Factors such as animal model and size, administration route, and number of administrations can also impact the necessary EVs dosage. Furthermore, these discrepancies hinder direct comparability between studies. Commonly used metrics for reporting EVs dosage include protein content, particle concentration, and cell equivalence (Table 4) [114].

Protein quantification of EVs is commonly performed using standard colorimetric assays such as the bicinchoninic acid (BCA) assay and the Bradford assay, which measure the concentration of vesicular proteins. However, these methods are primarily applicable to highly purified EVs samples, as the presence of contaminants from culture media or serum can lead to overestimation of protein content.

Particles numbers is typically estimated through techniques like nanoparticle tracking analysis (NTA) and tunable resistive pulse sensing (tRPS) [115]. NTA quantifies particles by capturing time-lapse recordings of their Brownian motion. This is done by imaging the particles using either scattered light or emitted fluorescence. By tracking numerous individual particle movements, NTA can estimate both the concentration and size distribution of particles. However, in complex biofluids, scattered light can interfere with accurate quantification by introducing artifacts from nonvesicular scattering sources, such as protein aggregates [116]. tRPS, in contrast, detects individual nanoparticles by monitoring variations in electrical current that occur when each particle passes through a tunable nanopore. While tRPS allows for high-resolution single-particle analysis, its application to minimally processed biological samples remains challenging due to the heterogeneity of EVs populations, which may result in nanopore blockage by larger vesicles. Furthermore, precise measurements require calibration using a buffer identical to that of the sample.

EVs dosage can also be expressed based on the number of parent cells from which they were derived. However, this method remains an approximation, as it does not account for temporal variations in vesicle production among individual cells.

Table 4 summarizes the EVs dosages used in various studies for different NDs, detailing the MSC source, experimental model (in vitro or in vivo), route of administration, and treatment regimen.

### 3.4. Source of MSC

The function of EVs is closely linked to their cargo, which can include functional mRNA, miRNA, lipids, and proteins. This composition is largely determined by the source of the cells and the physiological state of EVs originating cells [117, 118]. MSCs can be derived from various human tissues, with BM, AT, and UC being the most commonly utilized sources [119]. Moreover, more recent research has explored MSCs derived from additional tissues, including articular cartilage, brain, DP, skin, blood, and amniotic fluid (AM) (Table 4) [120].

A recent review article reported that BM-MSCs were the predominant source of EVs to treat brain diseases, accounting for 65% of the 74 publications included in the systematic review, followed by AT-MSC (14%) and placenta-derived MSC (14%). Similarly, in the context of spinal cord and peripheral nervous system diseases (50 publications analyzed in the systematic review), BM-MSC remained the most frequently utilized source (50%), followed by placenta-derived MSC (18%) and AT-MSC (16%) [114]. EVs secreted from different MSCs such as BM, AT, UC, and embryonic tissue are proved to promote angiogenesis and tissue repair [117]. When comparing EVs derived from UC-MSCs and BM-MSCs, both exhibits nearly identical physical properties and cargo composition, particularly concerning transcripts associated with immunomodulation and cell proliferation. The most notable distinction is that UC-MSCs generate EVs at a higher rate [121].

No studies in the literature have investigated how the source of MSCs may influence the production of EVs with superior therapeutic potential for acute or chronic NDs. To obtain EVs with optimal therapeutic efficacy for the treatment of a specific NDs, while addressing challenges such as low EVs yield, MSCs senescence, therapeutic dosing, and variability in MSC sources, preconditioning emerges as a highly effective approach.

## 4. Preconditioning Stem Cell Strategies to Improve EVs Properties

**Figure 2:**
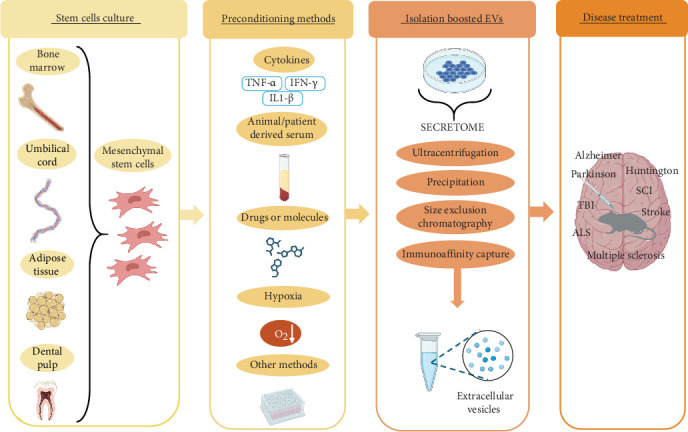
Overview of the preconditioning methods for MSCs derived from various sources to produce EVs for the treatment of NDs.

Preconditioning methods, such as three-dimensional cultures, exposure to hypoxia, and the utilization of various biochemical signals, have been shown to enhance the biological efficacy of MSCs-EVs, as these vesicles reflect the metabolic state and functionality of their parent cells (Figure 2) [122]. Notably, these approaches increase efficacy without compromising, and in some cases, even enhancing overall EVs production.

In this section, we present an overall of the heightened therapeutic effectiveness of EVs released by MSCs, using various preconditioning methods, in the treatment of the ND (Table 5).

### 4.1. Hypoxia

Hypoxia preconditioning of MSCs exposes cells to low oxygen levels, making them more resilient and therapeutically potent for transplantation. Capitalizing on the ability of MSCs to adapt to and influence the microenvironment in damaged or inflamed tissues, this approach can improve various cellular functions, such as stemness, immunomodulation, and the secretion of beneficial factors like exosomes. Hypoxia mimics the microenvironment that MSCs may encounter in tissues undergoing injury or inflammation. It is widely accepted that the oxygen tension experienced by cells within the body is lower than the atmospheric level of 21% [123]. The physiologically relevant levels of oxygen vary across tissues, ranging from approximately 12% in blood to as low as 1% in deep cartilage tissue. Within the BM, oxygen tension fluctuates between 4% and 7%. Hypoxia involves an oxygen concentration in the cellular environment of 1% [123].

#### 4.1.1. AD

The effect of hypoxia (1% O_2_) on hMSCs derived from various tissues, such as adipose, BM, and Wharton's jelly, varies [123]. Hypoxia effects on stemness, proliferation, immunomodulatory markers (IDO, PGE-2, and HLA-G), and exosome biogenesis markers (CD63, ALIX, TSG101, and Rab27 a/b) were studied. There was a significant increase in CD63 mRNA expression (greater than 20 ± threefold change) for hBM-MSCs and hWJ-MSCs after 6 h of hypoxia exposure, while hASCs were found to be least responsive. Hypoxia preconditioning significantly enhanced the exosome secretion by all tissue-specific MSCs, which showed differential response for expression of exo markers at the transcription level; however, when evaluated for protein expression, it was found that most of the marker's expression peaked between 12 and 24 h of hypoxia exposure. During this period, HIF-1 α expression was also elevated in all tissue-specific MSCs, indicating a correlation between hypoxia and exosome biogenesis and secretion pathways.

These results are in agreement with those of Liu et al. [124] and Cui et al. [125] which underscore the promising role of hypoxia preconditioning in enhancing the therapeutic efficacy of MSCs-derived exosome. Liu et al. [124] reveal that hypoxia-pretreated mice ASCs derived exosomes exhibit enhanced therapeutic effects compared to untreated counterparts, showcasing their ability to mitigate apoptosis, inflammatory cytokines, and cognitive impairments in AD models. They identify circ-Epc1 as a crucial contributor to these effects, demonstrating its regulation of TREM2 expression and its role in modulating microglial phenotypes. In vivo experiments demonstrate partial rescue of AD-induced cognitive impairment with circ-Epc1-containing ASCs exosomes, emphasizing their potential in alleviating AD-related cognitive deficits. Cui et al. [125] showed that exosomes from hypoxic mice BM-MSCs improve learning and memory function, reduce amyloid plaque deposition, and modulate inflammation in AD brains. The study also highlights the up-regulation of miR-21 and miR-181 c in hypoxia-preconditioned exosomes, suggesting their potential contribution to cognitive improvement.

#### 4.1.2. PD

The therapeutic potential of exosomes derived from hASCs was also investigated in mouse models of PD [140]. These hASCs-exo, derived from hASCs under hypoxic conditions, promoted angiogenesis in vitro in human brain microvascular endothelial cells and home to injured sites in the brains of PD model mice induced with MPTP. The intraperitoneal injection of hASCs-exo in PD animals model reduces the aggregation of α-SYN, increases the number of tyrosine hydroxylase (TH)-expressing positive cells, and improves dopamine production compared to the MPTP group without exosome treatment. The molecular mechanisms underlying the pro-angiogenic effects pass through the activation of the protein kinase A (PKA) signaling pathway and upregulation of vascular endothelial growth factor (VEGF) expression [161].

#### 4.1.3. SCI

Exosomes derived from hypoxic preconditioned hBM-MSCs suppressed neuroinflammation, promoted microglial polarization from pro-inflammatory M1 to anti-inflammatory M2 phenotypes, and improved functional behavioral recovery in a mouse model of SCI [126]. The miR-216a-5 p was identified as a key mediator in promoting microglial M2 polarization and neurological recovery after SCI. Similarly, Yang et al. [127] found that hypoxic EVs were more effective than normoxic EVs in promoting motor function recovery, apoptosis inhibition, and inflammatory response reduction after SCI in rats. They also identified miR-21 as a key factor in promoting the transformation of A1 astrocytes (proinflammatory phenotype) into A2 astrocytes (anti-inflammatory phenotype) by activating the JAK2/STAT3 signaling pathway.

The miR-511-3 p, enriched in EVs from hypoxic hASCs, demonstrated a protective role in regulating the TRAF6/S1P/NF-κB pathway and promoting neuronal survival after SCI [128]. Moreover, miR-511-3 p can suppress inflammation and apoptosis in PC12 cells. In vivo experiments showed that EVs from hypoxic hASCs (hypo-Exo) can improve the functional recovery of SCI rats. Mu et al. [129] explored the potential of hypo-Exo encapsulated in a peptide-modified adhesive hydrogel and transplanted for promoting angiogenesis and functional recovery in SCI therapy. The hydrogel, modified with the adhesive peptide PPFLMLLKGSTR, facilitated the local release of exosomes, leading to increased VEGF expression and improved angiogenesis. Both in vitro and in vivo experiments demonstrated significant functional recovery after treatment with hydrogel-encapsulated hypo-Exo.

Shao et al. [130] identified lncGm37494 as a key mediator in hypoxia-treated ASCs-derived exosomes, which suppressed the expression of inflammatory factors via modulation of miR-130b-3p and PPARγ expression, facilitating SCI repair.

Yuan et al. [131] investigated the therapeutic effects of pericyte-derived exosomes (PDEs) on SCI, emphasizing their role in improving blood supply, ameliorating endothelial function, protecting the blood-spinal cord barrier (BSCB), and promoting functional behavioral recovery in mice. In contrast, Luo et al. [132] showed a potential downside of injured neuronal exosomes, which accelerated transplanted MSCs apoptosis after SCI. However, they also highlighted the protective effects of hypoxic preconditioning on MSCs survival mediated by HIF-1 α, a protein that protects cells from stress. The hypoxic preconditioning could increase the survival rate of MSCs after transplantation.

#### 4.1.4. TBI

Chen et al. [138] explored the therapeutic potential of exosomes derived from both untreated ASCs and ASCs subjected to hypoxia preconditioning in the context of TBI. ASCs hypoxic derived Exo significantly reduced hippocampal nerve injury following TBI, leading to cognitive function recovery. An abnormal expression of circ-Scmh1 in ASCs hypoxic derived Exo was found. Circ-Scmh1 enhances the therapeutic and neuroprotective effects of Exos, promoting M2 microglial polarization and reducing inflammation-induced hippocampal nerve injury after TBI. Liu et al. [139] investigate the neuro-regenerative potential of 3D-printed collagen/silk fibroin scaffolds loaded with hypoxia-induced exosomes derived from hUC-MSCs using beagles. The goal is to address the challenges of brain defect cavities resulting from TBI. The researchers find that these scaffolds significantly inhibit apoptosis, reduce neuroinflammation, and promote the expression of markers associated with nerve fibers, myelination, axons, neurons, and synaptic connections in the injured brain. Moreover, the scaffolds enhance motor function recovery after TBI.

#### 4.1.5. Stroke

sEVs derived from hBM-MSCs cultured under hypoxic conditions showed distinct angiogenic properties compared to EVs derived from hBM-MSCs cultured under normoxic conditions [133]. Hypoxic MSCs-derived EVs promoted proliferation, migration, and tube formation of cerebral microvascular endothelial cells, leading to enhanced microvascular remodeling and neurological recovery in an IS model. Interestingly, this proangiogenic effect was dependent on the presence of polymorphonuclear neutrophils (PMNs), highlighting the intricate interplay between MSCs-derived EVs and immune cells in promoting angiogenesis after stroke. Similarly, Yu et al. [135] found enhanced neuroprotective effects of hBM-MSCs and CM-hBM-MSCs on microglia cells exposed to oxygen-glucose deprivation/reperfusion (OGD/R) injury. CM-MSCs alleviated microglial injury and apoptosis, promoting an anti-inflammatory phenotype and enhancing cell viability. Moreover, mice BM-MSCs-EVs can protect neonatal mice from stroke [136]. These findings indicated that IN administration of mBM-MSCs-EVs attenuated neuroinflammation by suppressing microglial activation and the accumulation of inflammatory cytokines and chemokines in ischemic-reperfused regions, providing protection against sub-acute injury after stroke. Also, exosomes from ASCs improved cognitive function by reducing neuronal damage in the hippocampus after cerebral infarction [134]. The exosomes delivered circ-Rps5, which regulated the expression of SIRT7 and miR-124-3p. These molecules promoted the shift of microglia from an M1 to an M2 phenotype, which helped to protect neurons and improve cognitive function. Kuang et al. [137] identify a novel mechanism in vitro by which ASCs-EVs exert neuroprotection and enhance neurological recovery following stroke. ASCs-EVs inhibit ischemia-induced autophagy by transferring miR-25-3p to neurons. The transferred miR-25-3p interferes with the p53/BNIP3 signaling pathway, contributing to the observed reduction in autophagy.

### 4.2. Cytokine

The stimulation utilizing pro-inflammatory cytokine is a strategy to enhance the anti-inflammatory properties of MSCs [162, 163]. It is noteworthy that such approaches not only enhance the anti-inflammatory abilities of MSCs but also lead to the release of highly immunomodulatory EVs. These EVs effectively target inflammatory and oxidative processes in various pathological conditions, acquiring the capacity to shift macrophages from the M1 to M2 phenotype by transporting microRNAs that regulate macrophage polarization. Therefore, the immunomodulatory characteristics of MSCs-derived exosomes may not be inherent but instead induced by the inflammatory microenvironment.

IN administration of EVs, derived from cytokine-preconditioned hBM-MSCs (TNF-α + IFN-γ, 24- or 48-h), was able to induce immunomodulatory and neuroprotective effects in triple-transgenic 3xTg-AD mice [141]. MSCs-EVs reduced the secretion of IL-6 and IL-1β, which are upregulated in AD brains while enhancing the secretion of IL-10—which induces the M2c polarization state (the “deactivated” phenotype)—associated with the formation of neuronal synapses.

Priming of mice BM-MSCs with IFN-γ has enhanced their immunosuppressive capacity, IFN-γ is associated with an overexpression of specific miRNAs, suggesting its possible role in the immunomodulatory action of MSCs. miR-467f and miR-466q present in their derived EVs modulate the pro-inflammatory phenotype of activated N9 microglia cells and of primary microglia acutely isolated from late symptomatic SOD1^G93A^ mice, a murine ALS model, by downregulating TNF-α and IL-1β expression [142].

Preconditioning hASCs with TNF-α and IFN-γ also alters the composition and efficiency of the secretome, thereby, influencing the outcome of perinatal asphyxia. When this preconditioned secretome is IN administered to rat pups exposed to asphyxia, it effectively suppresses long-lasting hippocampal oxidative stress, induces nuclear E2-related factor (NRF2) nuclear translocation, and reduces NRF2 protein levels in the cytoplasm. Additionally, it elevates levels of the antioxidant protein NQO1. The improvement in behavioral development, including righting reflex, negative geotaxis, cliff aversion reflexes, motor coordination, locomotion, anxiety, and recognition memory, further highlights the positive impact of this treatment [143].

Preconditioning hBM-MSCs with IL-1β, an additional inflammatory mediator recognized for its ability to enhance the regenerative potential of MSCs-Exos, resulted in an augmentation of the anti-inflammatory properties of MSCs-Exo in astrocytes. This effect was mediated through the NRF2 signaling pathway [144].

### 4.3. Drugs or Molecules

In the context of preconditioning, one strategy involves treating MSCs with a variety of different chemical stimuli, such as drugs and molecules, to enhance the amount and affect the content of EVs secretion. This approach aims to fortify the resilience of MSCs-EV in the targeted area and increase their therapeutic potential [164].

Rat BM-MSCs were preconditioned with melatonin, a hormone known for its roles as a free radical scavenger, as well as its broad-spectrum antioxidant, anti-inflammatory, and anti-apoptotic effects [145]. Melatonin-primed MSCs-derived EVs administered in SCI-induced NRF2 KO mice through a single tail vein injection increased M2-like microglia/macrophage polarization by shuttling ubiquitin-specific peptidase 29 (USP29). This process subsequently led to the inhibition of NRF2 ubiquitination and degradation. The study also demonstrated that the decreased N6-methyl adenosine (m6A) modification resulted in the upregulation of USP29 at the mRNA level [145].

Repeated intralesional injection of EVs derived from mice, UC-MSCs preconditioned with curcumin in C57BL/6J male mice with induced SCI significantly reduced the secretion of pro-inflammatory cytokines IL-6 and TNF-α, suppressed inducible nitric oxide synthase (iNOS) expression, while increased the secretion of anti-inflammatory cytokines IL-4 and IL-10. This indicated a stronger anti-inflammatory effect of Cur-EVs in the treatment of SCI compared to EVs alone. Despite no difference being found in motor functional recovery between EVs and Cur-EVs treatments, Cur-EVs exhibited significant improvements in the cavity area and axonal regeneration in the injured spinal cords. This suggests a stronger impact on axonal regeneration, suggesting a highly effective treatment for repairing nerve tissue after severe spinal SCI [146].

Another study focused on SCI reported that a distinct molecule, berberine (Ber), exhibited similar effects in modulating inflammation. Ber is a naturally occurring isoquinoline alkaloid known for its diverse pharmacological effects, including anti-inflammatory, antioxidant, anti-apoptotic, neuroprotective, and multi-organ protective properties [151]. Unlike Curcumin, Ber was loaded directly into EVs rather than preincubated with MSCs. These Ber-loaded EVs, derived from hUC-MSCs, were combined with GelMA hydrogel and administered in a rat spinal cord left-sided hemi-section model. The study revealed that Ber-loaded EVs lead to a reduction in local pro-inflammatory factors such as IL-1β, IL-6, IFN-γ, and TNF-α. Additionally, Ber-EVs demonstrated the capability to inhibit fibroblast migration and proliferation in vitro. Through its robust regulation of the microenvironment, Ber-EVs substantially reduced fibrosis, thereby fostering neuronal neogenesis and axonal lengthening. Moreover, the study observed that endogenous neural stem cells (NSCs) migrated toward the site of injury after SPI [151].

Lithium (Li), commonly known as a mood stabilizer, has been recognized for its neuroprotective properties and its ability to enhance neurogenesis under experimental disease conditions, such as stroke [148]. EVs derived from BM-MSCs pretreated with lithium chloride (LiCl) were administered via IV injection in male C57BL6 mice subjected to MCAO. This study has shown that Li-EVs conferred neuroprotection and facilitated neurological recovery. The positive impacts of Li-EVs were attributed to a range of mechanisms, such as the reversal of peripheral immunosuppression and the inhibition of TLR4-dependent signaling pathways [148].

Neuroprotection activity and anti-inflammatory effects were also observed for treatment with EVs derived from mice BM-MSCs pretreated with NaHS, which is believed to be an exogenous H_2_S donor. H_2_S, an endogenous gas transmitter, plays a regulatory role in various physiological and pathological processes in the brain. H_2_S alleviates microglial activation and pro-inflammatory cytokine production, offering protection against cognitive dysfunction resulting from neuroinflammation [165]. The experimental model involved inducing neonatal hypoxia-ischemia in C57BL/6 J female mice, followed by the administration of rat mice BM-MSCs-EVs pretreated with NaHS through intracardial injection. Results indicated that H_2_S-EVs were effectively taken up by microglia/macrophages, leading to a reduction in their activation and promoting the differentiation of CD11b^+^/CD45^low^ and CD11b^+^/CD45^high^ cells toward a beneficial phenotype. The administration of H_2_S-EVs targeted the injured region of the ipsilateral hemisphere, and up-regulated miR-7b-5p expression was reduced by hypoxia-ischemia insult [147].

Another approach was observed for IN treatment of EVs derived from hASCs preconditioned with deferoxamine (DFX) in Wistar rat fetuses on the final day of gestation. DFX, an iron-chelating drug, is known for its ability to stabilize Hypoxia-Inducible Factor 1-alpha (HIF-1α) and enhance the expression of genes involved in cell migration [143].

The preconditioning with DFX increased HIF-1α, the number of anti-inflammatory cytokines, and the total antioxidant capacity of EVs-ASCs. Consequently, DFX enhanced the efficiency of ASCs secretome, leading to an improved outcome in cases of perinatal asphyxia (PA).

Interestingly, the secretome produced similar effects whether ASCs were preconditioned with DFX or TNF-*α* + IFN-*γ*. This suggests the potential for different preconditioning strategies to yield comparable positive effects, providing flexibility in the application of MSCs-derived secretome for mitigating the effects of PA [143]. As reported by Kang et al. [149], hASCs pretreated with endothelial differentiation medium (EDM) promoted angiogenesis in vitro. The study identified miR-31 having a crucial role in MV-triggered angiogenesis by targeting FIH1 in vascular endothelial cells, this mechanism influences the angiogenic potential of ASCs-derived MVs.

BDNF, a protein of the neurotrophin family, plays a fundamental role in supporting the survival, growth, and maintenance of neurons in the brain, but the clinical applications are limited by its large molecular weight. Rat BM-MSCs were pretreated with BDNF, and the Exo produced was administered, through tail vein injection, to Sprague–Dawley rats with a TBI model induced by the electric cortical contusion impactor (eCCI). BDNF-primed rMSCs-Exos exhibited superior effectiveness compared to regular rMSCs-Exos in promoting cell migration and inhibiting apoptosis, as confirmed through both in vivo and in vitro experiments. Notably, the levels of miR-216a-5p were significantly increased in BDNF-Exos, and this upregulation was associated with the inhibition of inflammation in PC12 cells. This approach provides a new perspective on the potential application of proteins, especially those facing difficulties in traversing the BBB, in the treatment of TBI [150].

### 4.4. Animal Brain Extract

Stimulating MSCs with extracts from diseased tissues to produce EVs is important to harness the therapeutic potential of vesicles in intercellular communication and modulation of tissue response, aiming to enhance the understanding and treatment of diseases. This pre-conditioning method more faithfully mimics the injured environment the cells would encounter, pointing to a specific disease-associated protective MSCs activation. Using extracts from diseased tissues allows for the customization of treatment based on the specific condition of the patient. Since EVs can carry disease-specific molecular information, this approach enables a more targeted and personalized response compared to generic cellular therapies.

To precondition hUC-MSCs, replicating the intricate microenvironment associated with stroke, brain tissue obtained from cerebral infarction was utilized [153]. This study demonstrated that, in comparison to exosomes from normal conditions, exosomes preconditioned with infarcted tissue exhibited enhanced promotion of vascular remodeling, neuron survival, and neurological function recovery in permanent MCAO Sprague–Dawley rats. After a stroke, infarct-preconditioned exosomes further reduce apoptosis, which may be the key reason why infarct-preconditioned exosomes promote cerebrovascular remodeling.

These results align with the study of Lee et al. [154], where hASCs were preconditioned with stroke-injured rat brain extract, and the impact of EVs extracted from the conditioned secretome (CS) was tested on ischemic brain injury in MCAO-rat models. These findings substantiate the hypothesis that the therapeutic effectiveness of MSCs is predominantly facilitated by the paracrine action of EVs. This hypothesis has already been corroborated by Xin et al. [155], who demonstrated that exosome-enriched fractions from MSCs exposed to MCAO brain extracts facilitated the transfer of miR-133b from MSCs to primary cultured astrocytes and neurons, resulting in neurite outgrowth and functional recovery after cerebral ischemia.

Santamaria et al. [152] showed that CS derived from murine BM-MSCs pre-conditioned in vitro in an AD environment, represented by brain homogenate from APP/PS1 mice, fully replicates multiple neuro-reparative activities exerted by implanted MSCs themselves. Prolonged treatments showed plaque reduction, lower AβO concentration, and reduced glial activation, leading to restored memory and a decreased A1 phenotype. Regular weekly IN administrations of mice BM-MSCs-CS significantly reversed neuropathology in 25-month-old APP/PS1 mice. This study also confirmed that MSCs conditioning strongly influences the therapeutic efficacy of the CS. Lack of cell preconditioning failed to promote the therapeutic activity of the secretome, and MSCs exposed to young WT mouse brain homogenate yielded MSCs-CS with no reparative effect in 25-month-old APP/PS1 mice.

Finally, brain tissue extracts from TBI rats were used to precondition hUC-MSCs. The resulting CS was stereotactically injected into the dentate gyrus of the hippocampus in a rat model of TBI and improved learning and memory post-TBI compared to the administration of normal CS, benefiting newborn cell survival. Injury brain extracts significantly up-regulated neurotrophins and growth factors compared to normal extract, making the injury-preconditioned CS a superior candidate. Furthermore, it stimulated hUC-MSCs to produce neurogenesis proteins, immunomodulating factors, and apoptosis suppressors [156].

### 4.5. 3D Culture

In vitro, 3D culture systems offer the potential to increase cellular physiology by facilitating more realistic native interactions among cells and between cells and the surrounding matrix. The physical and mechanical properties of the extracellular environment play a crucial role in regulating various cellular processes of MSCs. These processes include adhesion, proliferation, migration, and differentiation functionalities, which are influenced by cell-matrix adhesion and dynamic feedback mechanisms. The 3D culture method enhances the ability to replicate a microenvironment, mimicking more closely the in vivo conditions and improving the therapeutic potential of MSCs in various applications, including tissue engineering and regenerative medicine [118, 160, 166].

Zhang et al. [81] conducted a comparative analysis between the exosomes derived from a 2D and a 3D culture of hBM-MSCs when they were injected IV in Wistar rats subjected to TBI. The administration of Exo derived from 3D culture of MSCs using a collagen scaffold played a pivotal role in triggering the expression of angiogenesis and neurogenesis factors. This, in turn, led to a reduction in functional deficits, eased neurovascular remodeling, and suppressed the expression of inhibitory molecules associated with axonal growth. In contrast to the Exo-2D group, the exosomes derived from the 3D culture (3D-Exo) demonstrated a major suppressive effect on the activation of GFAP-positive astrocytes and CD68-positive microglia/macrophages. Furthermore, the 3D culture exhibited a substantial increase in the production of exosomes in comparison to the 2D culture. It is noteworthy, however, that neither the 2D culture nor the 3D-Exo approach showed any significant alteration in cortical lesion volume when compared to each other. This suggests that while the 3D culture and associated exosome production have a positive impact on functional outcomes and immune response modulation, they do not appear to influence the overall cortical lesion volume.

Son et al. [157] introduced an innovative 3D platform aimed at enhancing neuroplasticity in MCAO-Sprague-Dawley rats and C57BL/6J mice. The study utilized hWJ-MSCs, employing a spheroid culture technique and seeding them into a micro-patterned well system. The EVs were administered via tail vein injection. The research proved that, in comparison to traditional 2D cultures, the production and reproduction of EVs, including both the number and size of particles as well as EVs purity, were more consistently achieved across various lots from the same donor and among different donors when utilizing the 3D platform. The EVs exhibited a significant presence of different types of miRNAs associated with brain recovery, neurogenesis promotion, axonal growth regulation, T-cell differentiation, and immunomodulatory activity.

Several studies have investigated the effects of intranasally administered EVs derived from hBM-MSCs 3D cultures on animal models of AD, including nontransgenic mice and 5xFAD mice. These EVs showed enrichment in syntenin-1 and CD63, while CD9 and TSG were not as prevalent. The study also revealed that 3D aggregation culture may enhance the production of exosomes or sEVs compared to other methods. In mice treated with MSCs-derived EVs, there was a decrease in the colocalization between glial fibrillary acidic protein (GFAP) and amyloid plaques, indicating a potential modulation of neuroinflammatory processes. Specifically, in 5xFAD mice, treatment with hBM-MSCs-EVs led to a significant reduction in cognitive decline and amyloid plaques in the hippocampus, accompanied by a reduction in Aβ levels throughout the rest of the brain [158]. They attribute these effects to the unique characteristics of EVs produced by 3D culture, which facilitate their passage across the BBB and modulation of neuroinflammation.

A similar outcome has been observed focusing on the cultivation of EVs from hUC-MSCs in a 3D graphene scaffold, exploring both the EVs content and its impact in APP/PS1 transgenic mice through ICV administration. Treatment with 3D-Exo markedly increased the expression of ADAM10 while decreasing the expression of BACE1; this led to a reduction in the generation of Aβ. Furthermore, the study observed significant expression of NEP, IDE, and HSP70 in the 3D-Exo group, suggesting enhanced Aβ degradation and inhibition of oligomerization. The findings revealed that the 3D graphene scaffold kept NSCs in a more active proliferation state compared to a 2D graphene film. Moreover, it promoted NSCs differentiation, particularly towards astrocytes and neurons.

Beyond Aβ-related mechanisms, 3D-Exo exhibited superior effects in mitigating inflammation, oxidative stress, and inhibiting microglial activity compared to 2D-Exo [159].

The latest approach to preconditioning with 3D culture, known as 4D culture, involves a three-dimensional culture that evolves over time, allowing it to mimic developmental scenarios and develop into homeostatic entities resembling critical tissue features such as functional cells and their ECM. For example, seeding NSCs in a sustained neural trophic factor (NT)-3 releasing bio-scaffold resulted in the formation of a self-organized neural network tissue with mature neuronal function [167], resulting in 4D-EVs. This innovative strategy has been employed in the treatment of rats experiencing compressive/contusive SCI.

The 4D culture technology showed the generation of a higher quantity of hUC-MSCs-EVs characterized by larger sizes and diverse proteomic profiles. Notably, there were substantial upregulations of epidermal growth factor receptor (EGFR) and insulin-like growth factor binding protein 2 (IGFBP2) in 4D-sEVs compared to their 2D counterparts. Upon endocytosis of 4D-sEVs, the binding of EGFR and IGFBP2 triggered downstream STAT3 phosphorylation and IL-10 secretion, helping the subsequent reduction in neuroinflammation at the injury site epicenter and resulting in significant neuroprotection, as showed by an increased number of surviving spinal neurons [160].

## 5. Conclusions and Perspectives

In conclusion, MSCs-derived EVs represent a promising cell-free therapeutic approach for the treatment of both acute and chronic NDs. Their inherent ability to modulate the immune system, promote tissue repair, and cross the BBB highlights their potential to address the complexities of neurodegeneration. However, challenges such as low EV yield, diminished therapeutic efficacy due to MSCs replicative senescence, and suboptimal targeting to injury sites remain significant barriers to their clinical application.

Preconditioning strategies have emerged as a viable solution to enhance the therapeutic potential of MSCs-derived EVs. By modulating the cellular environment before EVs isolation, these strategies can improve EVs yield, potency, and the therapeutic composition of their cargo. Various preconditioning modalities, including three-dimensional cultures, hypoxic conditions, and biochemical stimuli, have shown promise in optimizing the functional properties of EVs.

Despite these advancements, there is a clear need for more research focused on refining preconditioning protocols specifically tailored to neurodegenerative diseases. The current literature provides only limited insights into the effects of preconditioned EVs in both in vitro and in vivo models of NDs. Future studies should aim to establish standardized preconditioning techniques, assess long-term efficacy and safety, and explore the mechanistic pathways by which preconditioned EVs exert their neuroprotective effects.

Overall, while the field is still in its early stages, the potential of preconditioned MSCs-derived EVs to revolutionize the treatment of neurodegenerative diseases is undeniable. Continued research and development in this area could pave the way for innovative, effective therapies that significantly will improve the quality of life for patients suffering from these debilitating conditions.

## Figures and Tables

**Table 1:** Overview of research on the therapeutic potential of MSCs-derived EVs in AD, PD, and HD.

Disease	Treatment outcome				Reference
	Behavioral changes	Immune-related effects	Cellular and molecular effects	Neuroprotective/neurological effects	
AD	Enhanced spatial learning and alleviated memory decline	Elevated anti-inflammatory cytokines and reduced pro-inflammatory cytokines	Upregulated IDE and NEP, significantly reduced Aβ deposition, increased neuron viability, and reduced apoptosis	Promoted neurite growth and neurogenesis, decreased microglial activation, improved brain glucose metabolism, and slowed new plaque formation	[31–39]
PD and HD	Enhanced motor performance, reduced rotation defects, and delayed onset of symptoms	Mitigated neurotoxic effects and inflammation (PD)	Lowered apoptotic rates, boosted cell survival, encouraged cellular autophagy, activated mitochondrial function, and reduced aggregation of mHtt (HD)	Provided overall neuroprotective benefits, alleviated disease symptoms (PD), reduced striatal atrophy, decreased neuronal intranuclear inclusions, and improved neurological function	[40–42]

**Table 2:** Overview of research on the therapeutic potential of MSCs-derived EVs in MS and ALS.

Disease	Treatment outcome				Reference
	Behavioral changes	Immune-related effects	Cellular/molecular effects	Neuroprotective/neurological effects	
MS	Improved clinical symptoms, clinical score diminished, increased in body weight, and improved motor deficits	Modulated microglial polarization, suppressed pro-inflammatory responses in the CNS, decreased T-cell proliferation, and reduced leukocyte infiltration	Supported differentiation of oligodendrocyte precursor cells, reduced DNA damage, and preserved myelin in spinal cords	Promoted remyelination and protected oligodendrocytes	[44–52]
ALS	Improved motor performance and alleviated ALS symptoms	/	Reduced proapoptotic proteins, increased antiapoptotic proteins, decreased aggregation of mutant SOD1 proteins, reduced ROS production, protected mitochondrial membranes and restored their potential, and normalized p-CREB and PGC-1 α levels	Protected against lumbar motoneuron degeneration, restored oxidative phosphorylation, and enhanced complex I and ETS coupling efficiency	[53–58]

**Table 3:** Overview of research on the therapeutic potential of MSCs-derived EVs in stroke, TBI, and SCI.

Disease	Treatment outcome				Reference
	Behavioral changes	Immune-related effects	Cellular/molecular effects	Neuroprotective/neurological effects	
Stroke	Enhanced neurological recovery	Modulated peripheral immune response, maintained normal immune cell levels, reduced pro-inflammatory mediators, and avoided post-ischemic immunosuppression	Stabilized vascularization in the peri-infarct zone, inhibited astrocyte and microglia activation, and maintained BBB integrity	Significantly decreased infarct volume and promoted angiogenesis	[61–77]
TBI	Improved cognitive and behavioral performance and sensorimotor function	Decreased microglia-mediated inflammation and lowered pro-inflammatory cytokines	Reduced neuronal loss, cystinosis, and apoptosis and improved BBB integrity	Enhanced brain angiogenesis, neurogenesis, neural plasticity, and aided in tissue preservation	[78–87]
SCI	Enhanced locomotor function	Downregulated inflammasomes, pro-inflammatory factors, and activated microglia	Activated the ERK1/2 pathway to promote NSC proliferation and neural differentiation, reduced cell apoptosis, and decreased glial scar formation (lower GFAP expression)	Reduced tissue damage, promoted neural regeneration, increased Nestin expression, and supported neural progenitor cell activation	[88–99]

**Table 4:** Summary of studies reporting MSC source, MSC-EV dosage, treatment regimen, experimental model, route of administration, and targeted NDs.

MSC source	EV dose (protein)	EV dose (cells)	EV dose (particles)	Treatment regimen	Route administration	In vitro/in vivo model	Reference	NDs
hUC	30 μg/100 μL	—	—	Four doses	TV	AβPPswe/PS1dE9	[31]	AD
hUC	50 μg/150 μL	—	—	Daily (1 month)	TV	APPswe/PS1dE9	[32]	AD
mBM	22.4 μg/4 μL	3 × 10^6^	1 × 10^9^	Single	ICV	APPswe/PS1dE9	[33]	AD
hAT	1 mg/kg animal	—	—	Every 2 days (2 weeks)	IN	APPswe/PS1dE9	[34]	AD
mBM	30 μg/100 μL	—	—	Once/month (2 months)	ICV	SD-AD induced	[35]	AD
rBM	100 μg/500 μL	—	—	Single	IP	Albino rat-AD induced	[36]	AD
WJ	50 μg	—	—	Once/week (4 weeks)	IV	J20 (JAX-006293)	[37]	AD
iPSC	30 μg	—	—	Every 2 week (3 times)	Intracisternal	C57BL/6J-AD induced	[39]	AD
hUC	100 μg/500 μL	—	—	Every 3 days (8 weeks)	TV	SD-PD induced	[40]	PD
hUC	50 μg/5 μL	—	—	Single	ICV	C57BL/6J - PD induced	[41]	PD
hAT	200 μg/mL	—	—	Incubation 5 days	Culture media	NSCs from R6/2 mice	[42]	HD
mkBM	—	—	5 × 10^10^	2 times/week (4 weeks)	TV	C57BL/6-EAE	[44]	MS
rBM	100/400 μg	—	—	Single	TV	SD–EAE	[45]	MS
hPL	24 μg	—	—	Single	TV	C57BL/6-EAE	[46]	MS
hUC	50 μg	—	—	Single	IV	C57BL/6-EAE	[47]	MS
mAT	5 μg/300 μL	—	—	Single	IV	C57BL/6-EAE	[49]	MS
hAT	60 μg	—	—	Single	IV	C57BL/6-EAE	[50]	MS
hPL	—	—	1 × 10^7^/1 × 10^10^	Single	TV	C57BL/6-EAE	[51]	MS
mAT	10 μg	—	—	Daily (13 days)	TV	C57BL/6-EAE	[52]	MS
mAT	0.2–0.4 μg/mL	—	—	Incubation 2–18 h	Culture media	NSC-34 Cells (+oxidative damage)	[53]	ALS
mAT	0.2 μg/mL	—	6–8 × 10^5^ /mL	Incubation 6 h	Culture media	NSC-34 Cells (+oxidative damage)	[54]	ALS
hAT	200 μg/mL	—	—	At day 2, at day 6	Culture media	G93A Neuronal cells	[56]	ALS
mAT	0.2 μg/mL	8 × 10^6^	—	Incubation 3 h	Culture media	NSC-34 SOD1 (G93A)	[57]	ALS
hAT	200 μg/kg animal	—	—	Single	IN	Wr-stroke induced	[61]	Stroke
mkBM	—	—	4 × 10^11^/kg animal	2 times (after 14 days)	IV	Rhesus monkeys- cortical injury	[62–64, 74–76]	Stroke
hBM	—	2 × 10^6^	—	3 and 5 days after MCAO	IV	C57BL/6 - MCAO	[65]	Stroke
rBM	100 μg/500 μL	—	—	Single	TV	Wr–MCAO	[67]	Stroke
hBM	5.48–7.84 μg/μL	—	1.5–3.8 × 10^11^/mL	1,3, and 7 days after MCAO	IV	SD–MCAO	[68]	Stroke
rAT	100 μg/kg animal	—	—	Daily (3 days)	ICV	SD–MCAO	[69]	Stroke
rBM	100 μg/500 μL	—	—	Single	IV	SD–MCAO	[70]	Stroke
rBM	—	—	0.5–1–2 × 10^10^/mL	Single	TV	SD–MCAO	[71]	Stroke
hUC	200 μg	—	—	Single	TV	C57BL/6- MCAO	[72]	Stroke
hBM	—	1 × 10^5^	—	Incubation 24 h	Culture media	SK-N-SH, SH-SY5Y cell lines	[73]	Stroke
hBM	—	2 × 10^7^	—	2 times (1 h, 4 days after UCO)	IV	Fetuses texel ewes-UCO	[77]	Stroke
hBM	—	—	10–25.6 × 10^9^	Single	IN	C57BL/6-TBI induced	[78]	TBI
hAT	20 μg	—	2 × 10^10^/mL	Single	ICV	SD-TBI induced	[79]	TBI
hMSC*⁣*^*∗*^	100 μg	—	—	Single	IV	Wr-TBI induced	[80]	TBI
hMSC*⁣*^*∗*^	100 μg/500 μL	—	3 × 10^9^	Single	TV	Wr-TBI induced	[81]	TBI
rBM	100 μg/500 μL	—	—	Single	TV	Wr-TBI induced	[82]	TBI
hUC	50 mg/kg animal	—	—	Single	IN	Wr pup–perinatal brain injury	[83]	TBI
hBM	100 μg	—	—	One dose weekly (1 month) biweekly (1 month) six doses total	IV	C57BL/6-TBI induced	[84]	TBI
hBM	—	—	1 × 10^13^	Single	IV	Swine-TBI induced	[85]	TBI
hBM	—	—	1 × 10^12^	Single	IV	Swine-TBI induced	[86]	TBI
rBM	30 μg/150 μL	—	—	Single	Retro orbital	C57BL/6-TBI induced	[87]	TBI
hEF	100 μg/200 μL	—	—	Single	TV	SD-SCI induced	[88]	SCI
mBM	200 μg	—	—	Single	TV	C57BL/6-SCI induced	[89]	SCI
rAT	10/50 μg	—	—	Single	TV	Wr-SCI induced	[90]	SCI
hUC	20/200 μg	—	—	Single	TV	C57BL/6-SCI induced	[91]	SCI
hUC	200 μg/mL	—	—	Single	TV	SD-SCI induced	[92]	SCI
hUC	2000 μg/50 μL	—	—	Single	Spinal cord inj	SD-SCI induced	[93]	SCI
hEF	—	—	1–5 × 10^9^	Two times (after 3 days)	IV	SD-SCI induced	[94]	SCI
hUC	1–3 μL/10 μL	—	—	Single	IT	Wr-SCI induced	[95]	SCI
pAT	300 μg/300 μL	—	—	Single	IT	Pot-bellied pigs-SCI induced	[96]	SCI
hAm	100 μg/1000 μL	—	—	Daily (3 days)	TV	SD-SCI induced	[97]	SCI
mBM	200 μg/3 μL	—	—	Single	Injured hind limbs	C57BL/6-SCI induced	[98]	SCI
rBM	40 μg	—	—	Every 2 days (3 times)	TV	SD-SCI induced	[99]	SCI

Abbreviations: Am, amniotic; AT, adipose tissue; BM, bone marrow; EF, epidural fat; ICV, Intracerebroventricular injection; IN, intranasal injection; IT, intrathecal injection; IV, intravenous injection; MCAO, middle cerebral artery occlusion; NSCs, neural stem cells; PL, placenta; SD, Sprague–Dawley; TV, tail vein injection; UC, umbilical cord; UCO, unilateral carotid occlusion; WJ, Wharton jelly; Wr, Wistar rats.

*⁣* ^*∗*^Unspecified.

**Table 5:** Summary of preconditioning methods.

Preconditioning method	MSC source	EV dose (protein)	EV dose (cells)	EV dose (particles)	Treatment regimen	Route administration	In vitro/in vivo model	Reference	NDs
Hypoxia									
1% O_2_	hBM-hWJ-hAT	—	—	—	—	—	—	[123]	AD
1% O_2_	rAT	—	1 × 10^9^	—	Monthly (2 months)	Inj*⁣*^*∗*^	AβPPswe/PS1dE9	[124]	AD
95% N_2_ and 5% CO_2_	rBM	150 μg/80 µL	5 × 10^5^	—	Biweekly (4 months)	LCV	AβPPswe/PS1dE9	[125]	AD
1% O_2_	hBM	200 μg/200 μL	—	—	Single	TV	C57BL/6-SCI induced	[126]	SCI
1% O_2_	rBM	100 μg/500 μL	—	—	Daily (3 days)	TV	SD-SCI induced	[127]	SCI
1% O_2_	hAT	200 μg	—	—	Single	TV	SD-SCI induced	[128]	SCI
1% O_2_	hUC	—	—	—	—	Implanted	SD-SCI induced	[129]	SCI
1% O_2_	mAT	200 μg/200 μL	—	—	Single	TV	C57BL/6-SCI induced	[130]	SCI
1% O_2_	mPeric.	20 μg/300 μL	—	—	Single	TV	ICR-SCI induced	[131]	SCI
1% O_2_	rBM	—	—	—	—	Inj*⁣*^*∗*^	SD-SCI induced	[132]	SCI
1% O_2_	hBM	200 µL	2 × 10^6^	—	Every 48 h (3 doses)	TV	C57BL/6- MCAO	[133]	Stroke
1% O_2_	mAT	—	—	—	Single	TV	C57BL/6- MCAO	[134]	Stroke
1% O_2_	rBM	—	1 × 10^6^	—	—	—	—	[135]	Stroke
—	rBM	1 µg/1 µL 5 µg/1 µL	1.7 × 10^6^	—	Single	ICV/IN	C57BL/6- MCAO	[136]	Stroke
0.5% O_2_	mAT	10 μg	2 × 10^6^	—	Single	FV	C57BL/6-MCAO	[137]	Stroke
—	mAT	100 μg	—	—	Single	—	C57BL/6-CCI	[138]	TBI
—	hUC	200 μg	—	—	—	Implanted	Beagle-CCI	[139]	TBI
1% O_2_	hBM	200 µg/mL	—	—	5 weeks with 2 weekly inj	IP	BALB (c) mice PD induced	[140]	PD
Cytokine									
TNF-α + INF-γ	hBM	300 μg/mL	7 × 10^6^	15 × 10^9^	Two inj in 18 h	IN	3xTg-AD	[141]	AD
INF-γ	mBM	—	7 × 10^6^	10 × 10^6^	Four inj in 8 days	IV and IP	C57BL/6 mice-EAE	[142]	ALS
TNF-α + IFN-γ	hASC	6 μg/16 µL	2 × 10^5^	—	—	IN	Wistar pups asphyxia	[143]	Stroke
IL-1β	hBM	-	-	—	—	IN	LPS-treated astrocytes	[144]	NDs
Drug or molecules									
Melatonin	rBM	200 μg	—	—	Single	TV	NRF2 KO-SCI induced	[145]	SCI
Curcumin	mUC	1 µg/100 µL	2 × 10^6^	—	Four times every 7 days	IL	C57BL/6-SCI induced	[146]	SCI
NaHS	rBM	100 μg	—	1.5 × 10^8^	Single	IC	Pregnant C57BL/6-HI	[147]	Stroke
Li	rBM	13.5 μg	2 × 10^6^	—	3	IV	C57BL/6-MCAO	[148]	Stroke
EDM	hAT	100 μg/mL	—	—	—	—	—	[149]	Stroke
BDNF	rBM	100 μg	—	—	Single	TV	SD-TBI induced	[150]	TBI
Berberine	hUC	—	—	—	Implanted	—	SD-SCI induced	[151]	SCI
DFX	hAT	6 µg/16 µL	2 × 10^5^	—	2 h and 7 days post-asp	IN	Wistar pups asphyxia	[143]	Stroke
Animal brain extract									
Mice brain homogenate	hBM	—	1 × 10^6^ (*CM*)	—	Single	IN	AβPPswe/PS1dE9	[152]	AD
Cerebral infarct tissue	hUC	80 μg	—	—	Daily (3 days)	TV	SD–MCAO	[153]	Stroke
Stroke rat brain	hAT	0.2 mg/kg animal	—	—	Single	CA	Rat–MCAO	[154]	Stroke
Stroke rat brain	rBM	—	3 × 10^6^	—	Single	TV	Wr–MCAO	[155]	Stroke
Brain extract/injured brain	hUC	—	—	—	Single	IC	Rat-lateral fluid percussion	[156]	TBI
Other methods									
3D	hBM	1 µg/1 µL or 5 µg/1 µL	1.7 × 10^6^	—	Daily (10 days)	IV	Wistar rats CCI	[81]	TBI
3D	hWJ	10 μg	2 × 10^6^	—	Single	TV	SD–MCAO C57BL/6J-PT	[157]	Stroke
3D	hBM	100 μg	—	—	—	IN	NT-5XFAD	[158]	AD
3D	hUC	200 μg	—	—	Biweekly (4 months)	ICV	AβPPswe/PS1dE9	[159]	AD
4D	hUC	200 µg/mL	—	—	Single	Spinal	SD rats-SCI induced	[160]	SCI

Abbreviations: AT, adipose tissue; BM, bone marrow; CA, carotid artery injection; CCI, controlled cortical impact injury; CM, conditioned medium; HI, hypoxia-ischemia; IC, intracisternal; ICV, intracerebroventricular injection; IL, intra-lesional injection; IN, intranasal injection; IP, intraperitoneal injection; LCV, lateral caudal vein injection; MCAO, middle cerebral artery occlusion; PT, photothrombotic stroke; SD, Sprague–Dawley; TV, tail vein injection; UC, umbilical cord; WJ, Wharton jelly; Wr, Wistar rats.

*⁣* ^*∗*^Unspecified.

## Data Availability

The data sharing is not applicable to this article as no new data were created or analyzed in this study.

## Nomenclature

6-OHDA: 6-Hydroxydopamine
AchR: Nicotinic acetylcholine receptor
AMPK: AMP-activated protein kinase
AMSC: Amniotic mesenchymal stem cells
ASC: Adipose stem cells
ASOs: Antisense oligonucleotides
Aβ: Amyloid beta
BACE1: Beta-site APP cleaving enzyme 1
BBB: Blood brain barrier
BDNF: Brain-derived neurotrophic factor
Ber: Berberine
BMEC: Brain microvascular endothelial cells
BM-MSC: Bone marrow mesenchymal stem cells
BSCB: Blood-spinal cord barrier
Cav-1: Caveolin-1
CDKN2B: Cyclin-dependent kinase 4 inhibitor B
CM: Conditioned medium
CNS: Central nervous system
CS: Conditionated secretome
Cur: Curcumin
DFX: Deferoxamine
EAE: Autoimmune encephalomyelitis
ECM: Extracellular matrix
EDM: Endothelial differentiation medium
EF-MSCs: Epidural fat mesenchymal stem cells
EGFR: Epidermal growth factor Receptor
ESCRT: Endosomal sorting complex required for transport
ETS: Erythroblast transformation specific
Exo: Exosomes
EXOtic: EXOsomal transfer into cells
FAD: Familiar Alzheimer's disease
GFAP: Glial fibrillary acidic protein
HDAC4: Histone deacetylase 4
HIF-1α: Hypoxia inducible factor-1 alpha
Htt: Huntingtin
ICV: Intracerebroventricular
IDE: Insulin-degrading enzyme
IFN-γ: Interferon gamma
IGFBP2: Insulin-like growth factor binding protein 2
IL-1β: Interleukin-1 beta
IN: Intranasal
iNOS: Nitric oxide synthase
IV: Intravenous
MCAO: Middle celebral artery occlusion
MHC: Major histocompatibility complex
MPTP: 1-Methyl-4-phenyl-1,2,3,6-tetrahydropyridine
MVB: Multivescicular bodies
MWM: Morris water maze
NEP: Neprilysin
NF-κB: Nuclear transcription factor kappaB
NHP: Non-human primate
NLRP3: Pyrin domain-containing receptor 3
NORT: Novel object recognition test
NPC: Neuronal progenitor cells
NRF2: Nuclear E2-related factor
NSC: Neural stem cells
NSC-34(G93A): G93A point mutation
OGD/R: Oxygen-glucose deprivation/riperfusion
PA: Perinatal asphyxia
p-CREB: CAMP response element-binding protein
PGC-1α: Peroxisome proliferator-activated receptor gamma coactivator 1-alpha
PMN: Polymorphonuclear neutrophilis
PMSC: Early-gestational chorionic villus (placenta)-derived mesenchymal stem cells
PRX1: Peroxiredoxin 1
ROS: Reactive oxygen species
RVG: Rabies virus glycoprotein
SHED: Stem cells from human exfoliated deciduous teeth
SN: Substantia nigra
SOD1 (G93A): Human SOD1 gene with G93A mutation
TGF-β: Transformig growth factor-beta
TH: Tyrosine hydroxylase
TJ: Tight junction
TLR4: Toll like receptor 4
TNF-α: Tumor necrosis factor-alpha
tPA: Tissue plasminogen activator
UC-MSC: Umbilical cord mesenchymal stem cells
USP29: Ubiquitin-specific peptidase 29
WJ-MSC: Wharton jelly's mesenchymal stem cells
α-syn: α-Synuclein.
